# Digital management of diabetes global research trends: a bibliometric study

**DOI:** 10.3389/fmed.2025.1620307

**Published:** 2025-10-14

**Authors:** Shaoqi Zhu, Hupo Bian, Jianfeng Zhan, Lin Ni, Lixia Huo, Jia Hu

**Affiliations:** ^1^Department of Endocrinology, The First Affiliated Hospital of Huzhou University, The First People’s Hospital, Huzhou, Zhejiang, China; ^2^School of Medicine & Nursing, Huzhou University, Huzhou, Zhejiang, China; ^3^Department of Radiology, The First Affiliated Hospital of Huzhou University, The First People’s Hospital, Huzhou, Zhejiang, China; ^4^Huzhou Key Laboratory of Translational Medicine, The First Affiliated Hospital of Huzhou University, The First People’s Hospital, Huzhou, Zhejiang, China

**Keywords:** diabetes mellitus, digital management, bibliometric analysis, CiteSpace, VOSviewer

## Abstract

**Background:**

The rapid development in the field of digital diabetes management has captured significant attention. However, a comprehensive quantitative synthesis of the literature in this field remains scarce. This study aims to systematically map the evolutionary trajectory and knowledge structure of global research on digital diabetes management from 2010 to 2024, and to identify emerging research fronts and opportunity gaps within the field. Based on the bibliometric findings, we propose actionable recommendations for stakeholders to bridge the gap between technological validation and real-world implementation.

**Methods:**

The Web of Science Core Collection (WOSCC) was searched for publications on digital diabetes management from January 1, 2010, to December 16, 2024. The information was then thoroughly examined. The analyzed data was visualized using CiteSpace 6.2.4, VOSviewer 1.6.20, the R program “bibliometrix,” and the literature analysis website.

**Results:**

A total of 1,284 eligible publications were extracted from 101 countries/regions, with the United States contributing the highest number of articles. Meanwhile, Diabetes Care was identified as the most cited journal among various journals in the field. By analyzing the extracted literature with keyword clustering, the research hotspots were mainly focused on the “digital divide,” “artificial intelligence,” and “digital health.” In addition, an analysis of keyword emergence shows that “patient education,” “self-management education,” and “life style intervention” represent the current research frontiers.

**Conclusion:**

Artificial intelligence has received widespread attention as an important research area and emerging research trend in diabetes digital management. In the foreseeable future, the research paradigm in the field of digital diabetes management is gradually shifting toward enhancing patient engagement and emphasizing comprehensive lifestyle interventions.

## 1 Introduction

Diabetes, a chronic non-communicable disease of global prevalence, is increasingly scrutinized due to its myriad complications and unfavorable prognosis (1). The Global Burden of Disease (GBD) 2021 estimates predict that diabetes will rank as the fifth leading cause of Disability Adjusted Life Years (DALYs) worldwide in 2022, with an anticipated rise to third place by 2050 (2). This trend indicates a growing global burden attributable to diabetes relative to other diseases over time. Research highlights a significant increase in the economic burden associated with diabetes, driven by rising rates of hospitalizations and comorbidities that directly impact diabetes-related costs (3). Consequently, there has been a concerted effort among clinicians to identify improved glycemic management tools through digital diabetes management aimed at enhancing long-term prognoses.

Digital management of diabetes encompasses various activities including monitoring patients’ conditions, modifying treatment plans, providing health education, and offering self-management support via digital technologies such as mobile applications, wearable devices, telemedicine platforms, and electronic health record systems (4–7). This approach delivers personalized medical services and health advice by collecting and analyzing data on blood glucose levels, dietary habits, exercise logs, medication usage, and additional relevant information. It also enables healthcare professionals to monitor changes in patients’ conditions more effectively while striving for optimal medical management.

The long-term management of diabetes requires not only stringent self-discipline from patients but also robust support from an effective healthcare system. The rapid advancement of information technology has positioned digital management models as a prominent area of research and an innovative practical strategy for controlling diabetes. The adoption rate of digital health solutions and telemedicine is accelerating swiftly; notably accelerated by the restrictions imposed during the COVID-19 pandemic (8).

Digital management encompasses a diverse array of modalities, ranging from the recording and analysis of blood glucose monitoring data through mobile applications to telemedicine platforms that facilitate seamless communication between healthcare providers and patients. These platforms also offer guidance on diagnosis and treatment, as well as diabetes risk prediction and personalized treatment plan formulation leveraging big data and artificial intelligence technologies (9). Such digital tools are anticipated to transcend the temporal and spatial limitations inherent in traditional diabetes management, thereby enhancing both the efficiency and quality of care. Furthermore, they aim to bolster patients’ self-management capabilities, ultimately leading to improved health outcomes.

The global burden of diabetes continues to escalate, while digital technologies have emerged as pivotal tools for enhancing the efficiency and accessibility of diabetes management. However, the rapid development, vast volume, and structural complexity of literature in this field make it challenging for researchers and policymakers to swiftly grasp its overall knowledge structure, evolutionary trajectory, and research frontiers. Traditional literature reviews face difficulties in extracting comprehensive and relevant insights from the enormous body of existing publications. In contrast, bibliometric analysis offers a robust approach to dissecting both quantitative and qualitative information within journal articles (10), and has proven effective in identifying emerging topics and research fronts across multiple disciplines (11, 12). Currently, there is a lack of a comprehensive and systematic bibliometric study focusing on the past 15 years (2010–2024) in this domain, hindering a macro-level understanding of technological pathways and shifts in research priorities. This study aims to fill this gap by leveraging scientometric tools to uncover the knowledge base, collaboration networks, evolution of hotspots, and future opportunities within the field, thereby providing a systematic and objective reference for future research, technology development, and policy-making.

## 2 Materials and methods

### 2.1 Extraction of citation data

**Figure 1:**
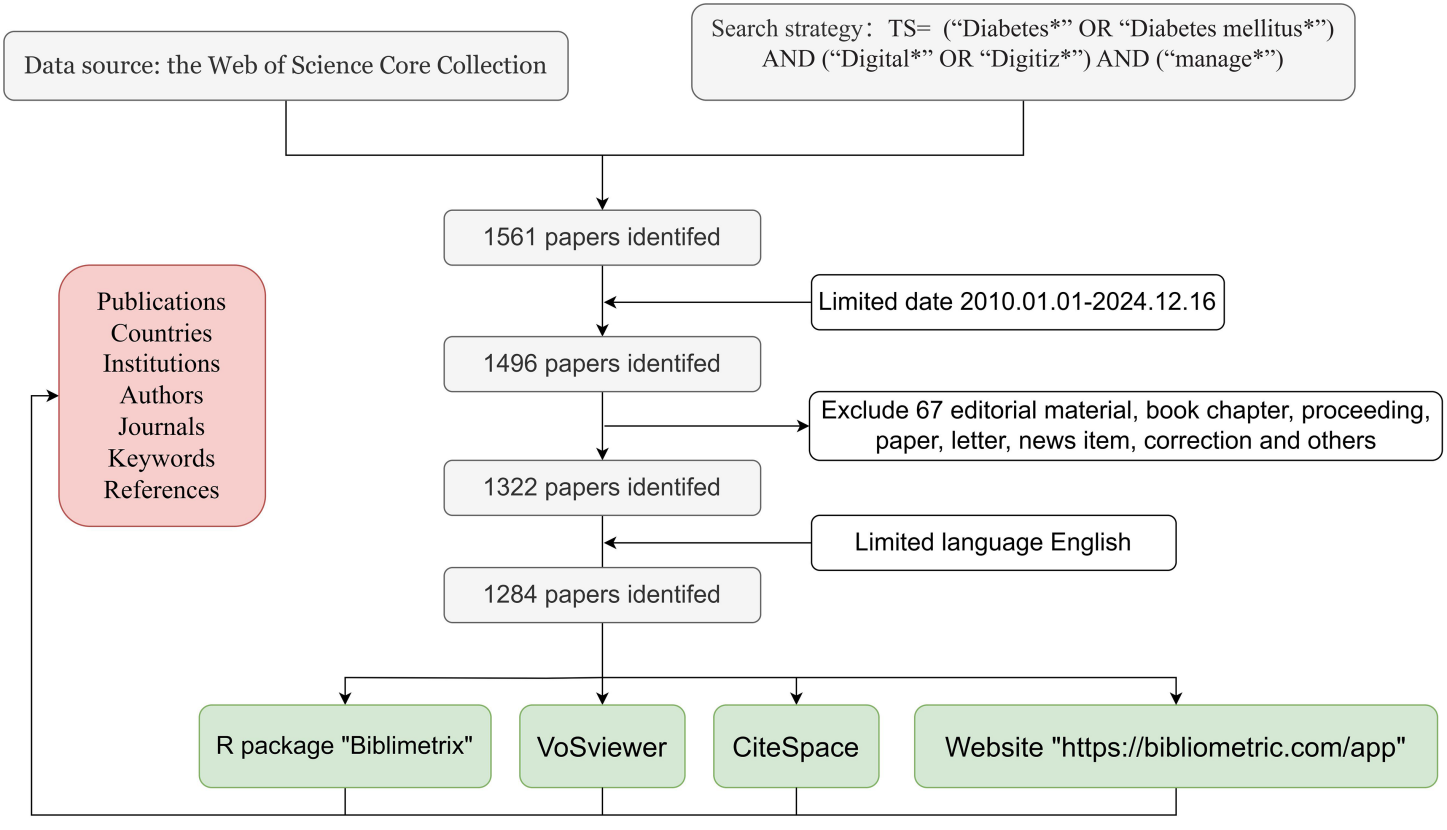
Framework diagram illustrating the screening and analysis methods used in the assessment of research literature related to the digital management of diabetes, 2010–2024.

We conducted a comprehensive search on 16 December 2024, selecting all papers published between 1 January 2010 and 16 December 2024 from the Web of Science Core Collection (WOSCC). The search was performed using the following formula: TS = (“Diabetes*” OR “Diabetes mellitus*”) AND (“Digital*” OR “Digitiz*”) AND (“manage*”). Articles and reviews written in English were considered. The initial screening process yielded a total of 1,284 original articles in English, including 976 articles and 308 reviews, which were considered potential candidates for inclusion in the study. For more detailed information on the literature extraction process, please refer to Figure 1.

### 2.2 Statistical analysis

The collected data of 1,284 documents were downloaded and selected as Full Record and Cited References, and the records were exported to Plain Text File by Export Records to Plain Text File to obtain the title, authors, abstract, keywords, and reference information of the documents, and then imported into the visual analysis software, VOSviewer1.6.20, CiteSpace6.2.4, R package “bibliometrix” and the literature analysis website: https://bibliometric.com/app. The output of annual publications, the relevant countries/regions, the institutions, most relevant authors, author output over time, journals, keywords, and co-cited references were used for quantitative and collaborative analyses. Subsequently, the journals were analyzed using CiteSpace with biplot overlays. Keywords and co-cited references were clustered, and timeline plots of co-cited references for keywords and publications were constructed to identify the top 25 keywords and references in terms of outbreaks, and the corresponding results were visualized. CiteSpace employs two metrics—Modularity (Q) and Weighted Mean Silhouette (S)—to evaluate clustering results based on network structure and cluster clarity. Modularity (Q) measures the interconnection density between modules, where Q > 0.3 indicates a significant community structure, with higher values denoting superior network partitioning. Weighted Mean Silhouette (S) assesses the quality of clustering techniques: S > 0.7 reflects highly convincing clustering efficiency, while S > 0.5 is considered reasonable (13).

## 3 Results

This study analyzed 1,284 publications originating from 7,311 authors at 2,755 institutions across 101 countries. These works were published in 501 journals and cited 49,541 references sourced from 13,632 distinct journals. The dataset comprised 976 research articles (76.01%) and 308 review articles (23.99%).

### 3.1 Publications

**Figure 2:**
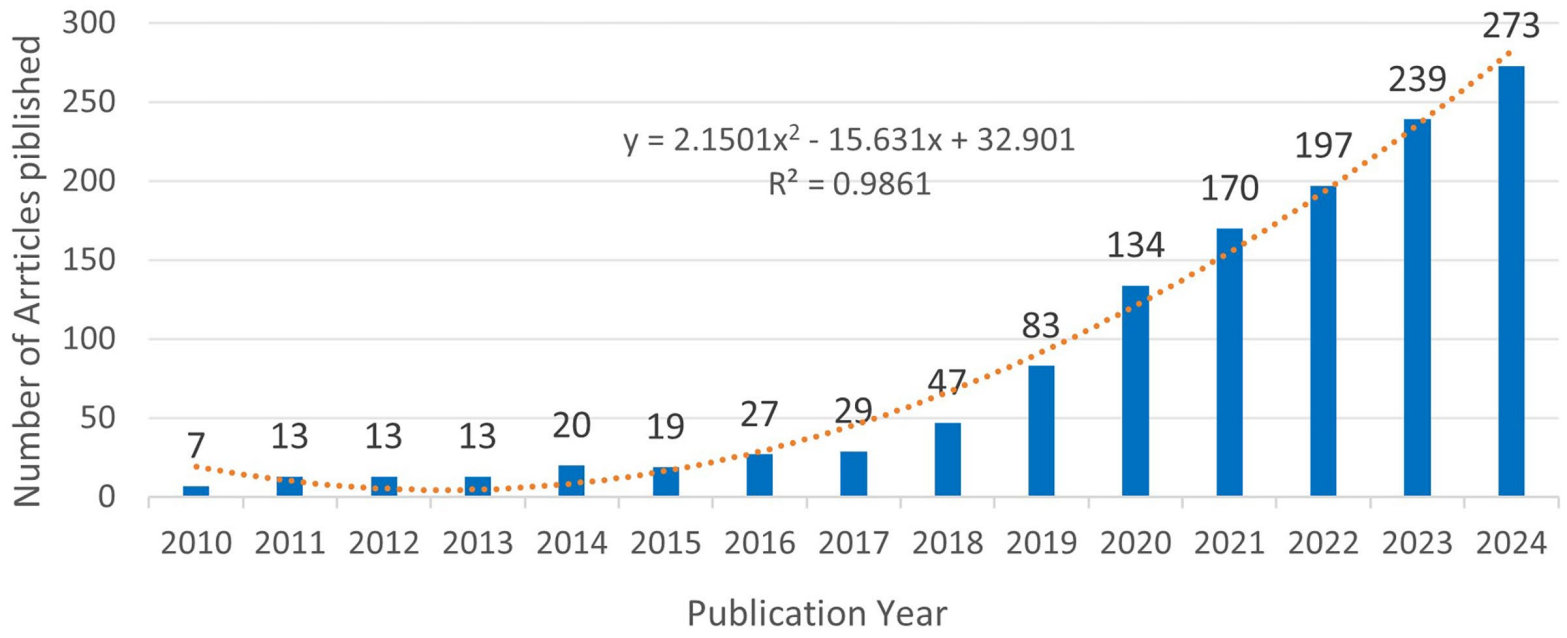
Trends in the number of publications on digital management of diabetes, 2010–2024. The equation y = 2.1501×2–15.631x + 32.901, where Y represents the yearly publication, and X represents the year. The coefficient of determination (*R*^2^) of the model was 0.9861.

Analysis based on the annual publication count, as depicted in Figure 2, reveals that from 2015 to 2018, the volume of publications concerning digital diabetes management consistently increased each year, culminating in a significant surge from 2019 to 2024. This trend indicates that research in digital diabetes management was a prominent focus during this timeframe, with a peak output of 273 articles recorded in 2024. In addition, to further understand the trend of the production, a polynomial fit to the trend line for annual publications was developed, the result suggesting a correlation between the year of publication and the number of annual publications, which is indicative of a continued increase in the number of digitally managed diabetes publications in the future. To determine whether the observed increase signifies a general rise in diabetes-related research or is specifically associated with digital management technologies, we conducted a separate search in the Web of Science Core Collection (WOSCC) under the categories “diabetes” and “digital management technologies.” We generated a trend analysis chart depicting the number of publications from January 1, 2021, to December 16, 2024 (Supplementary Figure 1), and fitted trend lines using standard polynomial regression. We analyzed publication trends from January 1, 2010, to December 16, 2024 (Supplementary Figure 1) and applied standard polynomial regression to fit trend lines to the annual publication count data. All coefficients of determination (*R*^²^) exceeded 0.9, indicating a good fit of the quadratic regression model to the observed data. Comparative analysis of Figure 2 and Supplementary Figure 1 reveals that the growth trajectory of publications on digital diabetes management aligns closely with that of digital management technologies, with both exhibiting a quadratic growth pattern characterized by an initial gradual expansion phase before 2018, followed by an accelerated growth phase thereafter. In contrast, while the broader diabetes field also follows a quadratic growth trend, its trajectory differs from these two digitally-focused domains. This convergence between the publication trends in digital diabetes management and digital management technologies supports the conclusion that digital management technologies play a pivotal role in driving research advances in digital diabetes management.

### 3.2 Countries and regions

**Figure 3:**
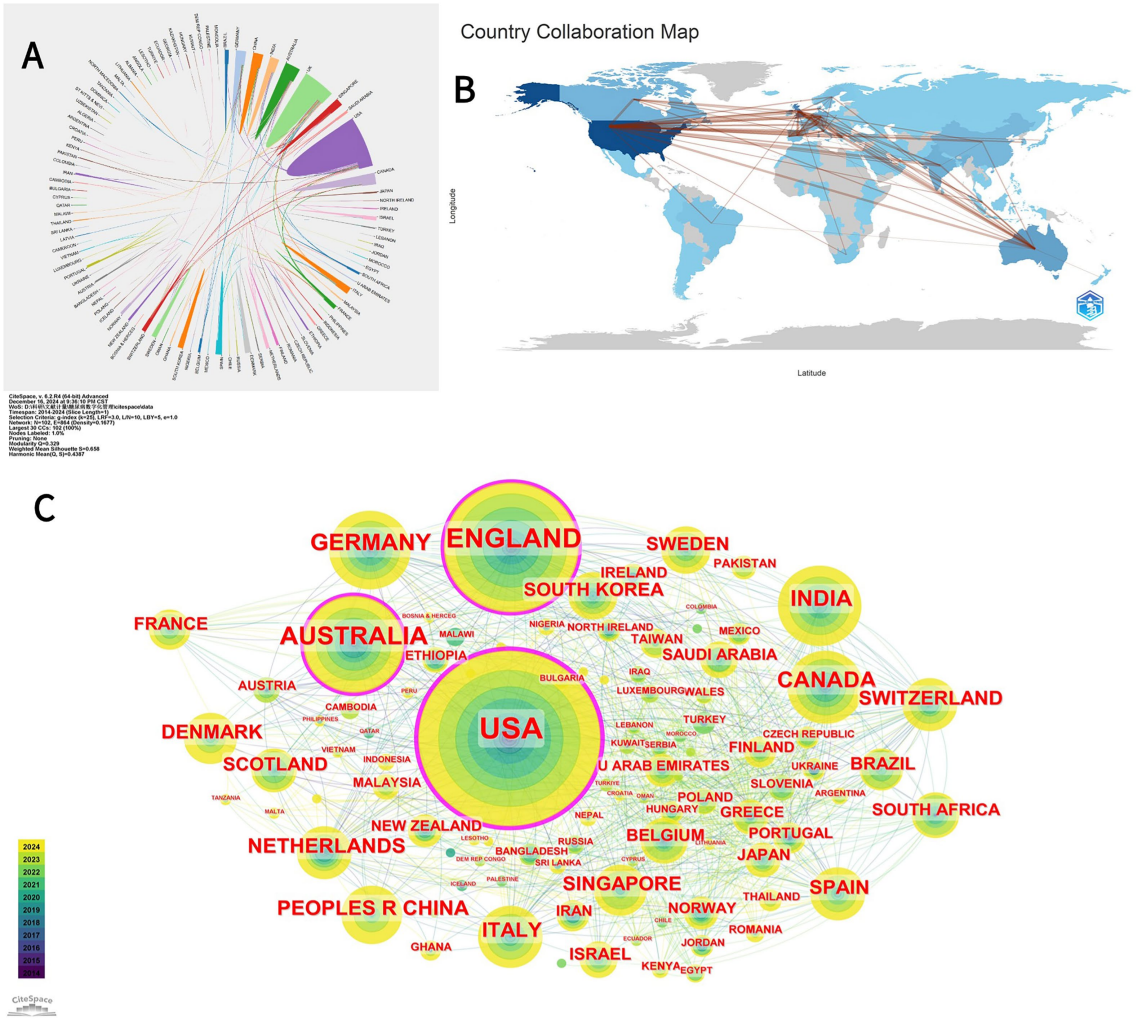
**(A)** A map illustrating bilateral cooperation networks among countries/regions regarding publications on diabetes digital management from 2010 to 2024. This map was generated using bibliometric analysis tools available at https://bibliometric.com/app; different color blocks represent various countries/regions, while color intensity indicates levels of collaboration between them. **(B)** Global collaborative map of countries/regions for publications on the digital management of diabetes from 2010 to 2024. The R package “bibliometrix” was employed for analyzing publication counts and collaborative activities by country/region. Darker blue shades indicate higher publication volumes per country/region; red lines denote collaborations between countries/regions, with thicker red lines representing stronger partnerships. **(C)** Collaboration map of countries/regions contributing to publications on the digital management of diabetes from 2010 to 2024. Generated using the bibliometric analysis software CiteSpace, each circular node represents a country/region; larger circles indicate a higher number of publications related to diabetes digital management in that specific area. The yellow and green segments denote different publication years (refer to the time color card in the bottom left corner for further details). The outermost purple circle identifies countries/regions with a centrality greater than 0.1, signifying those with significant influence in the field of diabetes digital management.

A total of 101 countries/regions have researched the topic of this paper. Figure 3A shows that the USA has published the largest number of articles (399), followed by the UK (222) and Australia (137). The top 10 countries in terms of output are summarized in Table 1, with the US having the highest centrality (0.32) and average number of citations per paper (39.3), significantly outperforming the other countries. The Netherlands has the second-highest average number of citations per article (36.2), but its centrality is relatively lower than that of the other countries. Figure 3B is a global map showing the collaboration between countries/regions around the world, and it can be seen that the US is more closely associated with several countries in Europe as well as with Australia. However, research on digital diabetes management remains disproportionately scarce across Africa and Southeast Asia. The international partnerships for each country are visualized using CiteSpace, as shown in Figure 3C, where the nodes represent countries, and the size of the nodes reflects the number of articles sent by the country. The purple part of the circle represents centrality greater than 0.1, and the United States, the United Kingdom, and Australia have higher centrality, indicating that they have frequent collaborations with other countries. In addition, the circle for the United States is the largest, indicating that this region has the most influential research in the digital management of diabetes.

**Table 1:** Top 10 countries or regions in terms of publications on digital management of diabetes, 2010–2024.

Rank	Country/Region	Count	Centrality	Average article citations
1	USA	399	0.32	39.3
2	ENGLAND	222	0.15	17.6
3	AUSTRALIA	137	0.11	14.7
4	GERMANY	92	0.1	14.5
5	INDIA	90	0.07	16.2
6	CANADA	78	0.04	19.1
7	PEOPLES R CHINA	58	0.01	11.6
8	ITALY	56	0.04	13.6
9	NETHERLANDS	51	0.09	36.2
10	SWITZERLAND	46	0.08	15.3

### 3.3 Institutions

Table 2 shows the top 10 most productive universities, with the University of California System, Harvard University, and, University of London being the top three universities with the highest number of published articles (103, 56, and 55, respectively). Notably that the University of California System is significantly more central than the other two universities (0.17), demonstrating its dominant influence in terms of publications. Five of the top 10 institutions are affiliated with the UK and four with the US.

**Table 2:** Top 10 organizations in terms of publications on digital management of diabetes, 2010–2024.

Rank	Institutions	Counts	Centrality	Countries or Regions
1	University of California System	103	0.17	USA
2	Harvard University	56	0.08	USA
3	University of London	55	0.13	ENGLAND
4	University of Oxford	36	0.12	ENGLAND
5	Johns Hopkins University	31	0.07	USA
6	University College London	30	0.09	ENGLAND
7	University of Manchester	27	0.04	ENGLAND
8	National University of Singapore	26	0.03	SINGAPORE
9	Imperial College London	25	0.11	ENGLAND
10	Duke University	20	0.04	USA

**Figure 4:**
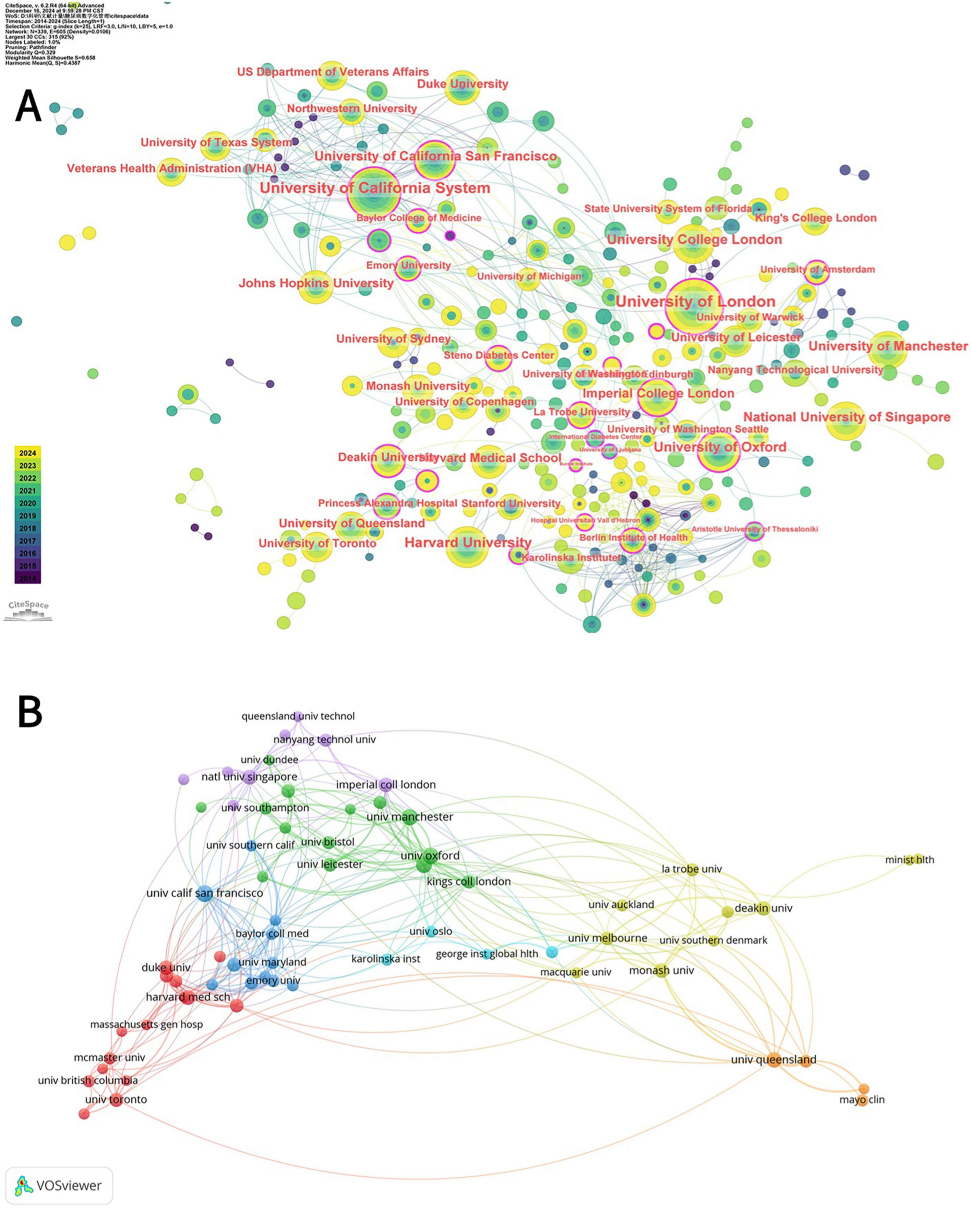
**(A)** CiteSpace network analysis of collaboration between institutions involved in the digital management of diabetes between 2010 and 2024. Each node with a colored chronology represents an institution, and the size of each node represents the relative amount of its research output. The different colors of the node ring represent the different years of publication (see the time color chart at the bottom left). The outermost purple circle is the institution with a centrality greater than 0.1, which represents the institution with a great influence in the field of diabetes digital management. **(B)** Superimposed visualization of the co-analysis of institutions involved in the digital management of diabetes from 2010 to 2024 conducted by VOSviewer. In the figure, institutions whose number of publications is greater than 8 are selected, and different nodes represent different institutions. Institutions with the same color have similar research directions, and the lines between nodes represent the cooperative relationship between institutions.

Collaboration between institutions is disclosed through the use of CiteSpace, as shown in Figure 4A. The connecting line between the two Figure 4B labels indicates close cooperation between organizations.

### 3.4 Analysis of authors

**Figure 5:**
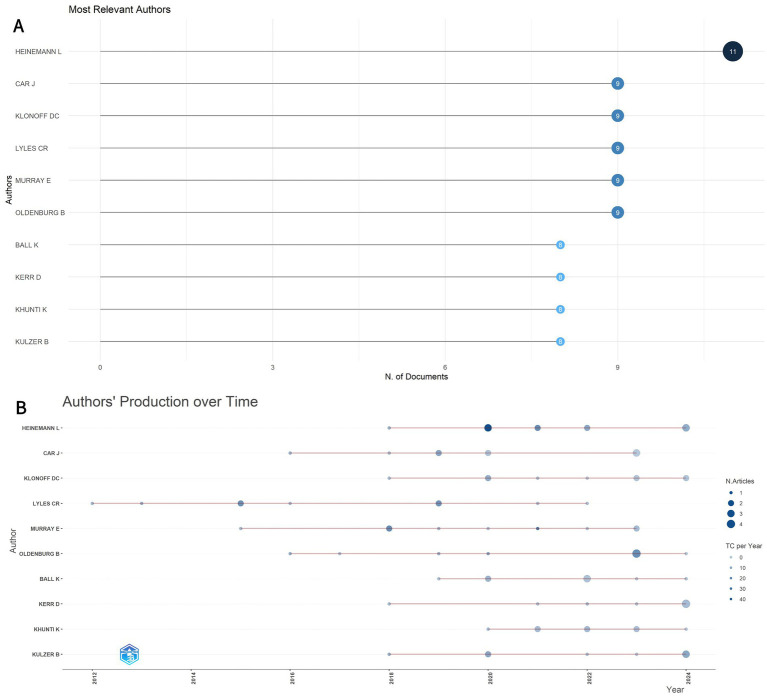
**(A)** The top 10 most relevant authors in the field. **(B)** Production of the top 10 authors over time. The size of the circle indicates the number of documents (N. Documents), and the color shading indicates the total number of citations (TC).

Figure 5A identifies the ten most relevant authors in this domain. At the author level, Figure 5B illustrates the evolving publication influence and annual output trends of these key contributors over the past decade. Among them, Heinemann L. demonstrates significantly high scientific impact. He is notably the corresponding author of the highly cited consensus report by the European Association for the Study of Diabetes (EASD) and the American Diabetes Association (ADA) Diabetes Technology Working Group, which reviewed challenges and recommendations related to digital health applications in diabetes (14). In contrast, Courtney R. Lyles has one of the longest research timeframes in this field, indicating an earlier entry into areas related to digital diabetes management.

### 3.5 Journals

By analyzing the cited and citing journals of the literature, it is possible to identify the influential journals in the field. Table 3 lists the top ten cited and citing journals, with the Journal Of Medical Internet Research ranking highest as the first citing journal, followed by the Journal Of Diabetes Science And Technology and Jmir Research Protocols. Among the cited journals, Diabetes Care took the top spot, followed by the Journal Of Medical Internet Research. In 2024, the Lancet had the highest impact factor among the cited journals, with an IF of 98.4, followed by the New England Journal Of Medicine, with an IF of 96.2.

**Table 3:** Top 10 citing and cited journals for publications on digital management of diabetes, 2010–2024.

Rank	Citing journals	Counts	Journal citation reports	2024 Journal Impact Factor
1	Journal Of Medical Internet Research	89	Q1	5.8
2	Journal Of Diabetes Science And Technology	43	Q2	4.1
3	JMIR Research Protocols	31	Q3	1.4
4	JMIR Formative Research	28	Q3	2
5	Digital Health	27	Q2	2.9
6	BMJ Open	21	Q1	2.4
7	BMC Health Services Research	20	Q2	2.7
8	JMIR mHealth and uHealth	18	Q1	5.4
9	Diabetes Technology & Therapeutics	17	Q1	5.7
10	Plos One	17	Q1	2.9
Rank	Cited journals	Counts	Journal citation reports	2024 Journal impact factor
1	Diabetes Care	2,989	Q1	14.8
2	Journal of Medical Internet Research	2061	Q1	5.8
3	JMIR mHealth and uHealth	956	Q1	5.4
4	Diabetes Technology & Therapeutics	942	Q1	5.7
5	Diabetic Medicine	872	Q2	3.2
6	Lancet	791	Q1	98.4
7	Journal of Diabetes Science and Technology	769	Q2	4.1
8	PLoS One	697	Q1	2.9
9	Diabetes Research And Clinical Practice	674	Q1	6.1
10	New England Journal Of Medicine	665	Q1	96.2

**Figure 6:**
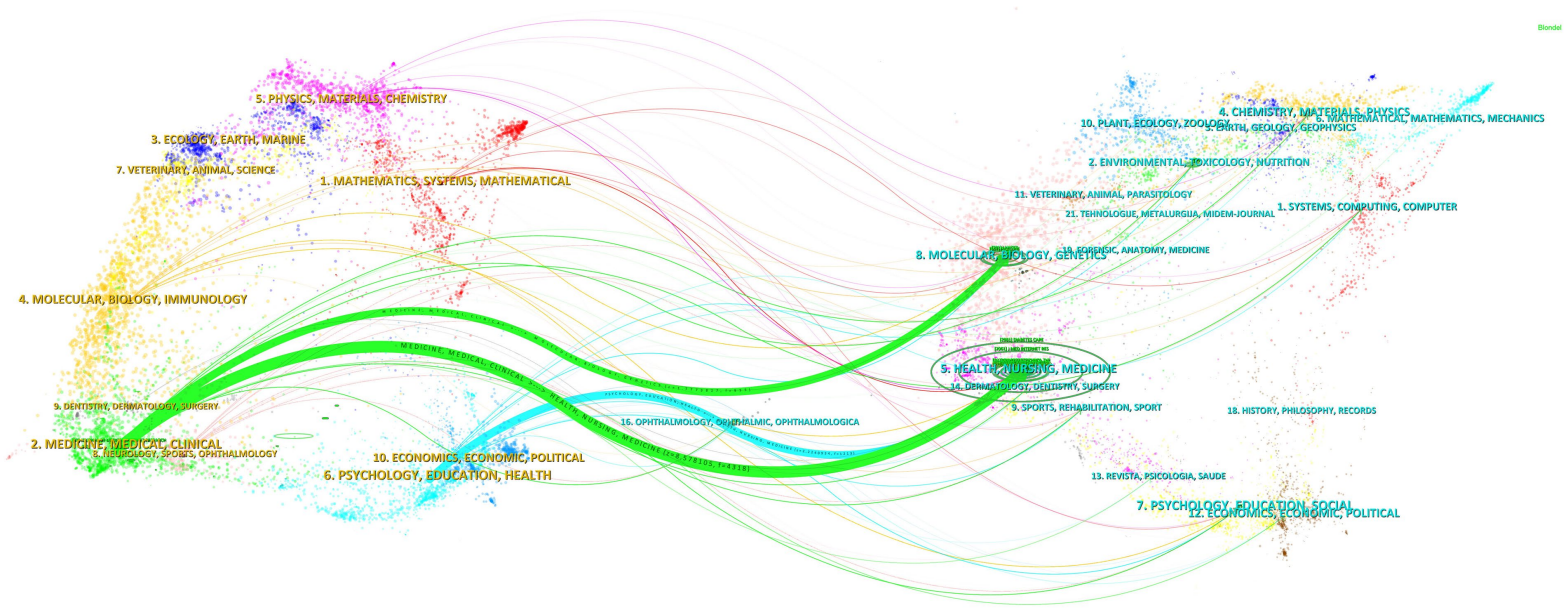
Dual-map overlay of journals in digital diabetes management, 2010–2024. Generated by CiteSpace.

In addition, we used CiteSpace to create a dual map overlay (Figure 6), analyzing journals that contributed to publications on the digital management of diabetes from 2010 to 2024. The clusters on the left are the citing journals, representing the knowledge frontier, and the clusters on the right are the cited journals, representing the knowledge base. Each label is centered on the clustering center of the corresponding journal and indicates the corresponding discipline in which the cited article was published. Each spline curve starts from the cited journal in the left bottom panel and points to the cited journal in the right bottom panel. As shown in Figure 6, it can be seen that the two most important citation paths (green) indicate that studies published in Molecular/Biology/Genetics and Health/Nursing/Medicine journals are mainly cited by literature in Medicine/Medical/Clinical journals, showing a confluent pattern. The other citation path (blue) shows that publications related to Health/Nursing/Medicine are also more likely to be cited by journals in Psychology/Education/Health.

### 3.6 Keywords

**Figure 7:**
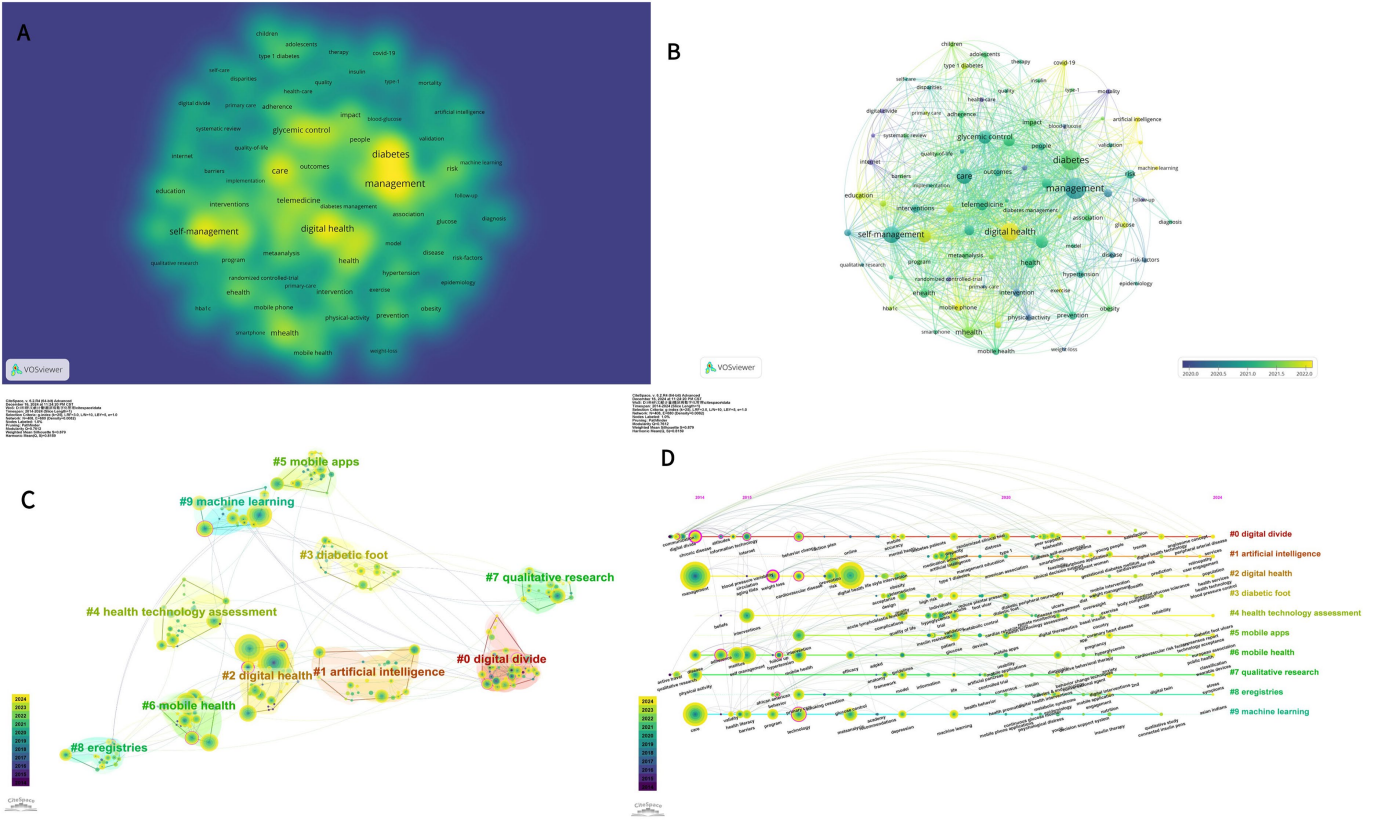
**(A)** Network and density visualization of co-occurring keywords in the digital management of diabetes from 2010 to 2024. **(B)** Network and Timeline visualization of co-occurring keywords in the digital management of diabetes from 2010 to 2024. **(C)** CiteSpace visualization of clustering views for keyword clustering analysis related to digital management of diabetes. **(D)** CiteSpace visualization timeline view of keyword cluster analysis related to digital management of diabetes.

In this study, we used VOSviewer to visualize 84 high-frequency keywords in the literature related to the topic of interest, setting a threshold of 25 occurrences for selecting these keywords. The resulting visualization in Figure 7A illustrates that color blocks adjacent to the center of the yellow blocks signify elevated citation and cited frequency, while the node size corresponds to the frequency of keyword occurrences. Figure 7B presents a network graph depicting the temporal distribution of the keywords, indicating the dynamics of crossover among various research domains.

Table 4 presents the ten most prevalent keywords ranked by frequency. Notably, “management,” “digital health,” and “care” emerge as the foremost keywords, signifying their prominence in this research domain over the past decade. The centrality scores for “glycaemic control” and “type 2 diabetes” are the highest, at 0.22 and 0.18, respectively, indicating their extensive interconnections with numerous other keywords and their role as critical links among various research topics or concepts. These concepts occupy a central position within the overarching knowledge network.

**Table 4:** Top 10 keywords related to digital management of diabetes, 2010–2024.

Rank	Count	Centrality	Keywords
1	255	0.06	Management
2	221	0.07	Digital health
3	171	0.04	Care
4	138	0.06	Diabetes mellitus
5	137	0.22	Glycemic control
6	130	0.18	Type 2 diabetes
7	125	0.06	Adults
8	116	0.03	Self-management
9	99	0.14	Health
10	91	0.01	Mellitus

The top 10 keyword clusters are generated based on the keywords; as shown in Figure 7C, the cluster “#0 digital divide” is the largest, followed by “#1 artificial intelligence “, “#2 digital health,” “#3 diabetic foot,” “#4 health technology. Figure 7D provides a timeline perspective illustrating the chronological description of the keyword cluster analysis. In Figures 7C,D, Modularity (Q) = 0.7612 and Weighted Mean Silhouette (S) = 0.879, indicating that the keyword clustering network is well-structured and the clustering results are convincing. Individual keywords are symbolized by nodes distributed along the time axis. The spatial layout of the nodes on the horizontal axis reflects the first appearance of the relevant keywords in the academic literature, giving a complete picture of the temporal evolution of the keyword clusters. The development of different research themes over time is presented, helping to identify the active and declining periods of each research theme.

**Figure 8:**
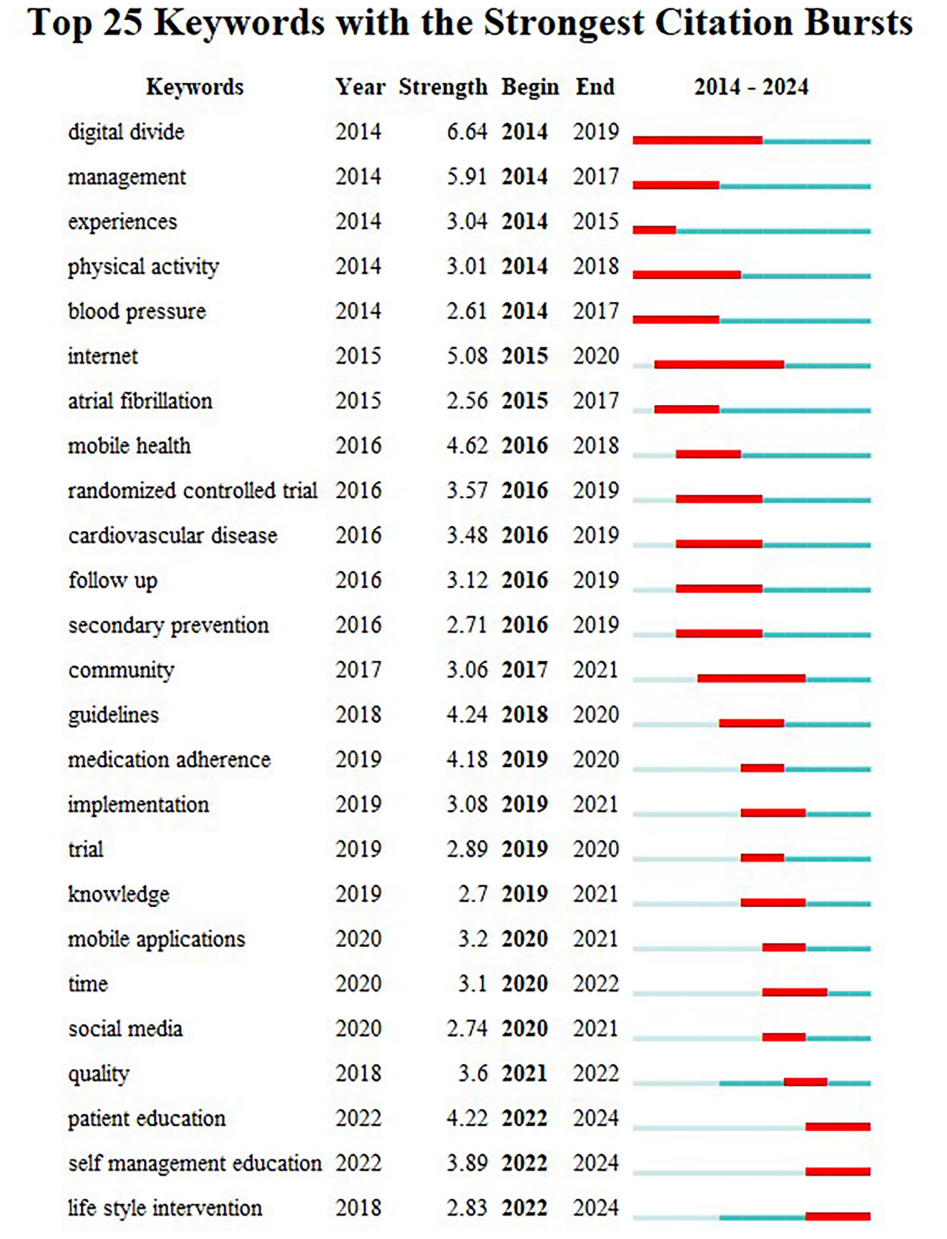
Keywords with the strongest citation bursts in publications on digital management of diabetes, 2010–2024.

CiteSpace identifies keywords with significant changes in frequency over a specific period, known as bursts. Keywords that appear late and last for a long period represent the latest research trends in a particular field, helping to retrace research hotspots and anticipate future trends. For the keyword burst detection analysis, burst strength serves as the metric quantifying the intensity of keyword emergence, which is directly proportional to the significance of frequency shifts. Higher burst strength indicates greater scholarly attention toward the keyword during the specified time period and a higher likelihood of representing research frontiers. We display the 25 strongest burst keywords, as shown in Figure 8. Between January 2010 and December 2024, the keyword with the highest burst intensity was “digital divide” (6.64), followed by “management” (5.91) and “internet” (5.9). “internet” (5.08). In the last 2 years, the keywords “patient education,” “self-management education,” and “life style intervention” have been floated. Of the three, ‘patient education’ had the greatest intensity of outburst at 4.22, suggesting that it currently represents a major research focus and may mark a pivotal moment with significant implications for future research.

### 3.7 Co-cited references

**Figure 9:**
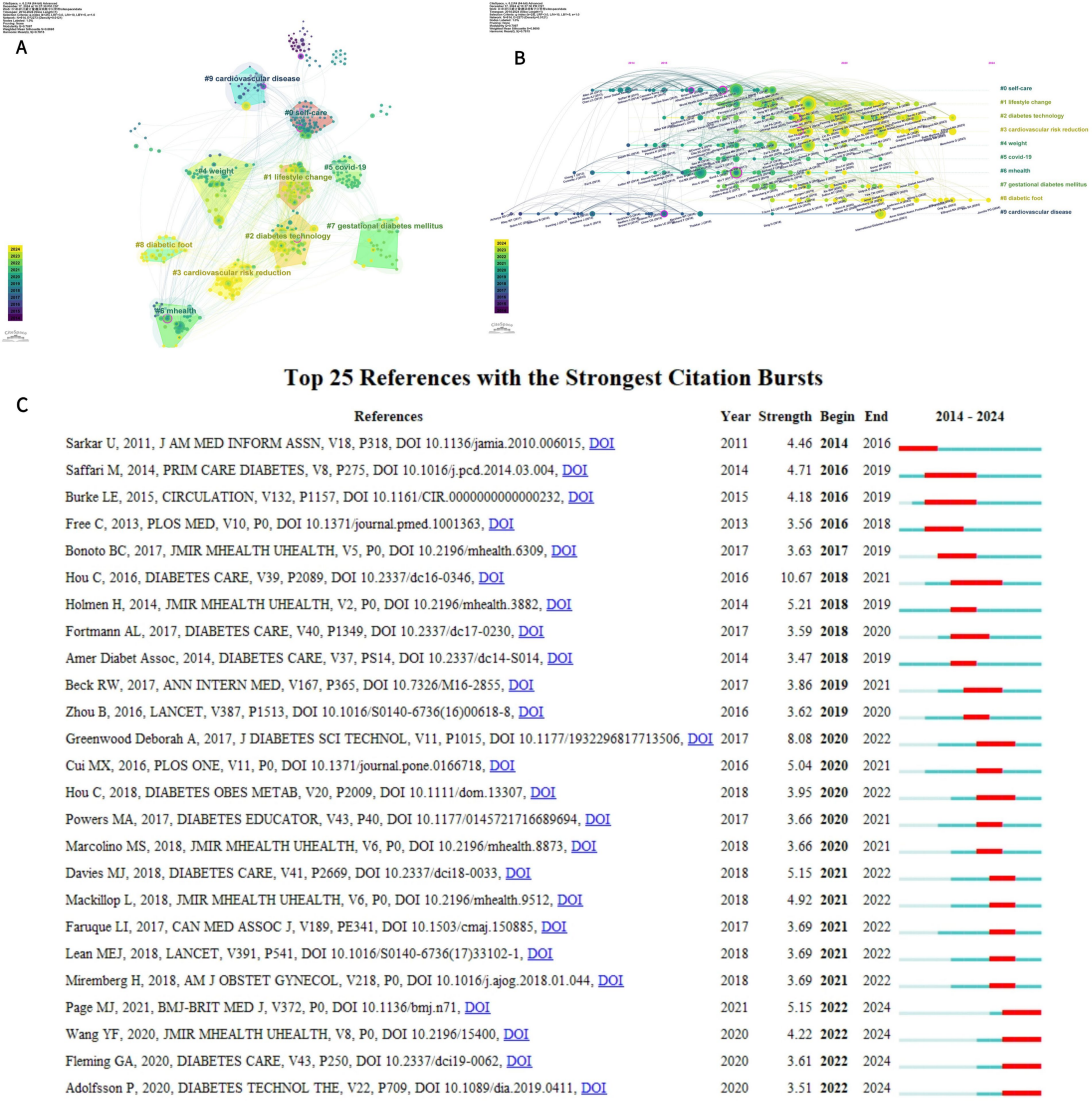
**(A)** Cluster analysis of co-cited literature related to digital management of diabetes visualized using CiteSpace **(B)** Timeline map of co-cited literature related to digital management of diabetes visualized using CiteSpace **(C)** Top 25 references of the 2010–2024 explosion.

A total of 49,541 references were cited, and the 10 most frequently cited references are listed in Table 5. A cluster diagram (Figure 9A) and a timeline diagram (Figure 9B) of the co-cited references were constructed using CiteSpace to understand the main research themes and their progress in the field. “#1 lifestyle change,” “#2 diabetes technology,” and “#3 cardiovascular risk reduction.” In Figures 9A,B, Modularity (Q) = 0.7097 and Weighted Mean Silhouette (S) = 0.8695, demonstrating that the co-citation clustering network is well-structured and the clustering results are reliable. It highlights the focus of research in the area of digital diabetes management. Meanwhile, Figure 9B depicts a timeline graph where the citation relationship between each reference is presented over time on the left timeline, where larger nodes indicate more frequent citations and node colors indicate when the reference was cited, highlighting the trend of research hotspots over time. Notably, the impact of digital management of diabetes on cardiovascular disease has been a prominent research hotspot since the early days. To conclude, Figure 9C shows a case study of 25 prominent references in the field, suggesting different directions of interest in the field over time, including “Do Mobile Phone Applications Improve Glycemic Control (HbA1c) in the Self-management of Diabetes? Diabetes? A Systematic Review, Meta-analysis, and GRADE of 14 Randomized Trials” was ranked first with an outbreak intensity of 10.67, reflecting a substantial impact on the field at that time (15).

**Table 5:** Top 10 publication references on digital management of diabetes, 2010–2024.

Rank	Count	Title of citing documents	DOI	Interpretation of the research
1	53	Digital health technology and mobile devices for the management of diabetes mellitus: state of the art (4)	10.1007/s00125-019-4864-7	As diabetes is difficult to manage and mHealth tools are emerging as useful, though not widespread, this paper reviews their interventions and points to The need to explore the impact of patient characteristics on efficacy and engagement.
2	40	A Systematic Review of Reviews Evaluating Technology-Enabled Diabetes Self-Management Education and Support (15)	10.1177/1932296817713506	A comprehensive review of literature from 2013 to 2017 encompassing 25 studies demonstrated that technology, including mobile phones, was useful for diabetes treatment; 18 studies indicated a drop in HbA1c levels, with communication, patient-generated health data, education, and feedback identified as critical factors.
3	36	Global and regional diabetes prevalence estimates for 2019 and projections for 2030 and 2045: Results from the International Diabetes Federation Diabetes Atlas, 9th edition (16)	10.1016/j.diabres.2019.107843	Analysis of 255 data sources from 1990 to 2018 revealed that the global prevalence of diabetes reached 9.3% in 2019, with higher rates observed in urban and high-income nations, alongside a substantial number of undiagnosed individuals and significant future growth.
4	34	Clinical Targets for Continuous Glucose Monitoring Data Interpretation: Recommendations From the International Consensus on Time in Range (22)	10.2337/dci19-0028	Despite the increased uptake of continuous glucose monitoring (CGM), its clinical utilization remains low. The 2019 ATTD Conference gathered specialists to provide consensus guidelines for the utilization and reporting of CGM data across various diabetic populations.
5	32	Diabetes Digital App Technology: Benefits, Challenges, and Recommendations. A Consensus Report by the European Association for the Study of Diabetes (EASD) and the American Diabetes Association (ADA) Diabetes Technology Working Group (13)	10.2337/dci19-0062	Digital health technology is evolving fast, diabetes apps are lagging in regulation, and a joint review by EASD and the ADA identifies problems and proposes measures to help them realize their potential. Proposes measures to help them realize their potential.
6	31	Do Mobile Phone Applications Improve Glycemic Control (HbA1c) in the Self-management of Diabetes? A Systematic Review, Meta-analysis, and GRADE of 14 Randomised Trials (14)	10.2337/dc16-0346	An examination of five libraries of studies from 1996 to 2015, encompassing 1,360 individuals across 14 studies, revealed that individuals with type 2 diabetes who utilized mobile phone applications for HbA1c reduction experienced more benefits, particularly among younger demographics.
7	26	Epidemiology of Type 2 Diabetes - Global Burden of Disease and Forecasted Trends (20)	10.2991/jegh.k.191028.001	A meta-analysis and multi-library search assessing the efficacy of mobile phone health applications for diabetes mellitus patients indicated that these applications can reduce HbA1c levels, assist patients in self-care and glycemic management, and enhance their confidence.
8	22	Efficacy of Mobile Apps to Support the Care of Patients With Diabetes Mellitus: A Systematic Review and Meta-Analysis of Randomised Controlled Trials (18)	10.2196/mhealth.6309	To assess the efficacy of mobile applications in managing patients with diabetes mellitus, 1,263 participants from 13 studies were analyzed across various repositories, revealing a significant reduction in HbA1c levels within the intervention group in 6 randomized controlled trials.
9	21	Management of Hyperglycemia in Type 2 Diabetes, 2018. a Consensus Report by the American Diabetes Association (ADA) and the European Association for the Study of Diabetes (EASD) (17)	10.2337/dci18-0033	The ADA and EASD collaboratively released a consensus on type 2 diabetes care, revising pharmaceutical guidelines based on new data, highlighting comprehensive therapy, individualized drug selection, and recognizing research deficiencies.
10	21	State of Type 1 Diabetes Management and Outcomes from the T1D Exchange in 2016–2018 (21)	10.1089/dia.2018.0384	Data on type 1 diabetes in the U. S. indicate that only a minority achieves management goals, adolescents and young adults exhibit inadequate control, and despite the heightened utilization of technology, there is no overall enhancement, alongside the persistence of racial inequities.

By analyzing the highly cited literature presented in Table 5, numerous studies have confirmed through randomized controlled trials and meta-analyses that mobile health technologies—such as diabetes management applications and continuous glucose monitoring (CGM)—can significantly reduce patients’ HbA1c levels. The mechanism underlying this effect is primarily based on real-time feedback from patient-generated data and remote interactions between healthcare providers and patients (4, 15–17). However, some studies reveal critical contradictions; despite a substantial increase in technology adoption (e.g., a threefold rise in CGM usage), the rate of blood glucose target attainment among adolescents and ethnic minorities remains stagnant. This stagnation underscores design flaws within digital tools regarding cultural appropriateness and support for health literacy (18, 19). International consensus documents further advocate for technical standardization, clarify the core clinical objectives of CGM, and establish an evidence-based evaluation framework for digital therapeutics (14, 20, 21). Additionally, the Global Burden of Disease study has quantified the prevalence of undiagnosed diabetes patients, providing an urgent impetus for regulatory innovation (18, 22). It is noteworthy that the “technology-efficacy disconnect” highlighted by T1D Exchange data (19) directly reflects the central challenge articulated by the joint statement from ADA/EASD—the lack of interoperability among medical systems impedes effective technology transfer (14). Collectively, these studies demonstrate that while the clinical value of digital diabetes management has been substantiated, its large-scale implementation necessitates urgent adaptations to personalized technology as well as comprehensive reconstruction of health service systems. The above analysis synthesizes key findings from Table 5 highly cited publications, highlighting major evidence trends and contradictions within the co-citation network.

## 4 Discussion

### 4.1 General information

This bibliometric analysis demonstrates that digital diabetes management has evolved from an emerging niche into a rapidly expanding, globally interconnected field. The exponential growth trajectory in publication output—distinct from the linear trend observed in general diabetes research—signifies that technological innovation itself serves as the primary driver of scholarly attention. Concurrently, the COVID-19 pandemic accelerated the advancement of digital diabetes research, as lockdown restrictions drove its transition from an adjunct tool to an essential care infrastructure (8). Geographically, research leadership remains concentrated in the United States, United Kingdom, and Australia, which function as collaborative hubs facilitating global knowledge exchange. However, the high centrality and citation impact in these regions contrast with the fragmented contributions from other areas, indicating persistent inequities in research capacity. This may potentially exacerbate the digital divide. The literature further exposes a critical translational gap: while robust evidence confirms the efficacy of digital diabetes management in improving clinical outcomes, such as reducing HbA1c levels (23, 24), real-world adoption is hampered by design limitations in health literacy support, cultural adaptability, and interoperable health systems (14). Consensus documents and meta-analyses increasingly advocate for standardized evaluation frameworks and patient-centered design—a necessary pivot to bridge the “technology-efficacy disconnect” (4, 14, 19).

### 4.2 Research hotspots

Keywords serve as critical indicators of research focus. The clustering of high-frequency keywords and the keyword map generated by CiteSpace reveal several central logically interlinked research domains that have shaped the field over the past decade including “digital divide,” “artificial intelligence,” and “digital health.”

The “digital divide” is the primary barrier to the advancement of digital diabetes management. There is a huge disparity in the accessibility and application of digital technologies among different geographic regions, socio-economic strata, and age groups around the world, with less economically developed regions and older patient populations often having a harder time enjoying digital health applications (25). Economically disadvantaged regions, exemplified by Southeast Asia and South Africa, experience “data poverty” due to insufficient health data generation (26–28). This scarcity impedes the broad implementation of data-driven digital health technologies (DHTs) and may exacerbate health inequities (29, 30). Root causes include infrastructural deficits, inequitable resource allocation, and cultural incompatibility (26). Theoretical perspective analysis indicates that this digital divide essentially constitutes a “socio-technical systems failure” jointly revealed by the Technology Acceptance Model (TAM) (31) and Health Equity Impact Assessment (HEIA) (32): TAM identifies dual core barriers for disadvantaged populations, where inadequate adaptation of digital tools to localized clinical needs compromises “perceived usefulness,” while interface complexity exceeding users’ digital literacy causes lack of “perceived ease of use” (33); HEIA further highlights structural resource allocation imbalances across three critical dimensions: (1) biomedical dimension manifested as insufficient comorbidity management support, (2) socioeconomic dimension reflected in the disproportion between device costs and household income, and (3) health system dimension evidenced by severely lagging digital infrastructure in primary care (34). To systematically address this multi-level challenge, establishing a technology-community-policy collaborative intervention system is required. In the technological innovation dimension, developing adaptive solutions aligned with TAM’s ease-of-use principles and HEIA’s economic accessibility principles is essential. Research demonstrates that SMS-based interventions meet “perceived ease of use” needs for low-digital-literacy populations through minimal interaction, demonstrating effectiveness in reducing HbA1c (35). Open-source mHealth applications directly address HEIA’s economic dimension, significantly reducing development costs and simplifying functions (36, 37). In the community implementation dimension, building a digital health ecosystem anchored to HEIA’s social equity goals is necessary. For instance, the Accountability, Coordination, and Telehealth in the Valley to Achieve Transformation and Equity (ACTIVATE) project enhances “perceived usefulness” via a community-co-designed remote monitoring platform, with training by community health workers significantly improving health outcomes for rural diabetic patients (31, 38). Given TAM’s revealed positive correlation between digital literacy and technology acceptance, targeted digital skills training programs are needed (36, 39). Additionally, digital health navigator programs implemented through community health centers effectively increase patient portal utilization rates (40). In the policy support dimension, implementing HEIA-oriented systemic reforms is critical. Governments should expand digital health infrastructure through funding, reducing healthcare cost barriers (41, 42), and establish cross-sector collaboration mechanisms integrating resources to address intersecting exclusion networks across economic-technological-geographical-cultural dimensions, particularly focusing on compounded marginalization challenges of vulnerable groups (25, 43). In summary, key stakeholders should take targeted actions: policymakers optimize resource allocation and cross-departmental cooperation; developers prioritize user-centered design principles; clinicians enhance digital literacy and localized support. Cross-sector collaboration remains crucial for achieving equitable adoption of digital health innovations.

As a pivotal technical component in the digital management of diabetes, artificial intelligence (AI) plays a transformative role in this domain (9), characterized by multi-level technology integration and clinical value. The application of AI can be categorized into four core areas: (1) risk prediction and early intervention: Multimodal models utilizing ensemble learning algorithms (such as XGBoost and LightGBM) amalgamate genetic information, clinical parameters, imaging data, and socioeconomic factors. These models are capable of predicting the risk of new-onset diabetes within 5 years (AUC 0.78–0.80) (44, 45) as well as assessing the likelihood of complications such as diabetic foot ulcers (DFU) and chronic kidney disease (CKD), with AUC values exceeding 0.85 (46, 47). (2) Precision screening and diagnostic optimization: Ikram and Imran (48) introduced a hybrid model named ResViT FusionNet that significantly enhances the accuracy of diabetic retinopathy (DR) detection and classification by leveraging the strengths of both convolutional neural networks (CNNs) and vision transformers (ViTs). Moreover, interpretable artificial intelligence techniques such as Local Interpretable Model-agnostic Explanations (LIME) and Gradient-weighted Class Activation Mapping (Grad-CAM) are employed to augment transparency and clinical interpretability within this model (48). Similarly, Suganthi et al. proposed a dual-track deep learning model that integrates the Swin Transformer with an efficient multi-scale attention-driven network (EMADN) for diabetic foot ulcer (DFU) classification. This approach enhances interpretability through Grad-CAM and achieves an accuracy of 78.79% along with a macro F1 score of 80% on the DFUC-2021 dataset, outperforming existing methods (49). (3) Personalized treatment decision: Automatic insulin delivery (AID) represents a novel therapy for blood glucose management within a closed-loop system comprising CGM, insulin pumps, and control algorithms (50). AI-enhanced decision support systems can optimize automated insulin therapy through advanced algorithmic capabilities (51). A six-month randomized multicenter trial demonstrated that AID utilizing an AI algorithm significantly increased the percentage of time blood glucose levels remained within the target range (70–180 mg/dL) compared to sensor-augmented insulin pumps, while also reducing glycosylated hemoglobin levels (52). By analyzing electronic health record (EHR) data, AI can provide clinicians with valuable decision support regarding antidiabetic drug treatments. Federico et al. (53) introduced a time-span guided neural attention model known as Tangle to accurately predict the necessity for second-line diabetes treatment following metformin failure, achieving an area under the ROC curve of 90%. (4) Comprehensive management of diabetes: The application of AI in comprehensive diabetes management primarily encompasses medical nutrition therapy and blood glucose monitoring. In terms of medical nutrition therapy, Fang et al. (54) proposed an innovative method based on generative adversarial networks (GANs) to estimate food energy values from images captured in mobile food records. Furthermore, integrated digital healthcare platforms designed for AI-driven dietary management have been shown to improve blood glucose control and facilitate greater weight loss outcomes (55). In the realm of blood glucose monitoring, AI technology can be employed to interpret biomedical data for patients and issue alerts, thereby facilitating improved control over their blood glucose levels (56).

Despite AI’s transformative potential in diabetes management, its clinical application continues to encounter several technical challenges and ethical dilemmas. At the data level, limited sample sizes and heterogeneous datasets—such as cross-institutional electronic health records—restrict the generalizability of models. Furthermore, the performance of clinical models may vary with geographic location and temporal factors; thus, it is essential to continuously update model parameters by integrating real-time data (57). Additionally, attention must be given to data privacy and security concerns. There exists a risk that algorithms could be intentionally hacked, leading to program errors that may harm numerous patients (58). At the algorithmic level, many algorithms exhibit embedded biases due to the underrepresentation of minority groups within datasets (59). This situation exacerbates existing digital divides. Consequently, confounding factors associated with relevant models require careful evaluation (60). AI serves as both a “regulator” and an “amplifier” of digital disparities; its ultimate impact hinges on the synergy between technological adaptation and systematic intervention strategies (61, 62). Moreover, the lack of clinical interpretability inherent in black-box models—particularly CNNs and multi-layer perceptrons—impedes physicians’ trust in these systems (63). The study reveals that AI, as a key enabling technology in digital diabetes management, demonstrates considerable potential across core domains including risk prediction, precision diagnosis, personalized treatment, and integrated care. However, its clinical adoption still faces multiple challenges such as limited data generalizability, algorithmic bias, privacy and security concerns, and insufficient model interpretability. To facilitate the transition of AI technology from the “validation phase” to “universal healthcare,” it is imperative to establish multi-center validation frameworks, develop low-cost edge computing solutions, and formulate coordinated governance strategies that integrate technical adaptation and systemic intervention. Researchers should focus on enhancing model transparency and fairness, policymakers must build regulatory systems that ensure equity and security, and patients and communities need to improve digital health literacy and technology accessibility. Only through multi-stakeholder collaboration can we foster AI-driven innovation and scalable implementation in diabetes management, ultimately enabling more equitable, effective, and sustainable healthcare services.

In the digital management of diabetes, the concept of digital health permeates all facets of diabetes care. DHTs encompass a broad spectrum of domains, including wearable devices, mHealth, health information technology, and telemedicine. The primary objectives are to enhance healthcare accessibility, reduce healthcare costs, improve efficiency in service delivery, and ultimately better patient outcomes through technological innovations (64). mHealth serves as a crucial mechanism for achieving these goals by utilizing voice calls, SMS messaging, wireless data transmission, and mobile applications to provide comprehensive healthcare services (65). Real-time collection and uploading of health data are facilitated through seamless connectivity with wearable devices. mHealth applications can assist individuals with diabetes in assessing and managing foot-related health issues by offering customized software for foot and ankle exercises as well as wound assessment tools while providing training on personal foot care practices and observation techniques (66). Similar technologies are also being employed to support self-care initiatives related to blood glucose control and the management of other complications associated with diabetes (67–69). Healthcare professionals remotely monitor patients’ health status in real-time and intervene in a timely manner in case of abnormal data, breaking the time and space constraints of traditional healthcare and building an all-encompassing health management ecosystem (70). Consistent with our findings, a recent systematic review by Zaki et al. (71) highlights the practical utility of digital interventions—including mobile apps, EHRs, and telehealth—in enhancing glycemic control among Type 2 diabetes patients. The widespread adoption of DHTs has significantly facilitated diabetes management; however, it may also exacerbate existing health inequities. For instance, patients residing in low-income or remote areas may be unable to fully leverage these technologies due to limited Internet access or a lack of digital devices (72), thereby intensifying the uneven distribution of health resources. Furthermore, DHTs involve the collection and processing of substantial amounts of personal health data, which are often gathered remotely. It is imperative that the confidentiality, integrity, and availability of this data are safeguarded against unauthorized access or disclosure (73). In this context, the study conducted by Kezbers et al. (74) effectively identified instances of false registration behavior in remote recruitment through the development of a deception detection program, providing a valuable practical reference for enhancing data security. Research into DHT applications remains at an early stage regarding both development and translation into practice. The market features a diverse array of DHT applications and devices that exhibit significant variability in quality and functionality (75–77). Consequently, there is an urgent need to establish unified evaluation standards and specifications for these technologies. Regulatory frameworks governing DHTs play a crucial role in their approval and adoption processes—not only to ensure that these technologies are safe and effective but also to promote equity and accessibility aimed at bridging the digital divide. This ensures that such technologies benefit all populations, particularly those who are vulnerable. For example, the U. S. Food and Drug Administration (FDA) has established a hierarchical approval system via its “Digital Health Pre-Cert Program,” which prioritizes the review of innovative technologies based on real-world evidence (RWE). This initiative enhances the efficiency of regulatory reviews concerning diabetes digital management applications (78). Additionally, under Section 1557 of the Affordable Care Act, regulations issued by the Office for Civil Rights (OCR) within the U. S. Department of Health and Human Services (HHS) prohibit discriminatory outcomes from patient care decision-making tools—including artificial intelligence—thereby promoting specific measures to mitigate algorithmic bias (79). Moreover, the European Medicines Agency (EMA) proposed and signed the European Declaration on Digital Rights and Principles in 2022. This declaration emphasizes a people-centric approach to promoting solidarity and inclusion, as well as freedom, security, and empowerment within the digital environment. It advocates for enhanced connectivity, digital education, training, fair working conditions, and accessible digital public services to foster participation and sustainable development in the digital public space (80). The clinical integration of AI and DHT necessitates a structured three-step approach: “technical verification, clinical adaptation, and system reconstruction.” Taking AI-enabled CGM as an illustrative example, this transformation process encompasses algorithm validation, the embedding of clinical workflows, and the reconstruction of prognosis evaluation systems (20). The incorporation of digital management with AI decision support has been shown to enhance the reduction of HbA1c levels in patients with type 2 diabetes (81).

Concurrent emergence and proliferation of specific keywords can illuminate trends in research hotspots. As illustrated in the keyword burst chart presented in Figure 8, the emerging keywords from 2022 to 2024 include “patient education,” “self-management education,” and “lifestyle intervention.” This trend reflects a reorientation and renewed focus within the field of digital diabetes management during this period. Early technology-driven studies focused on algorithm optimization and equipment accuracy were hindered by a lack of continuity in efficacy during practical applications. For instance, there was a notable disconnect between the popularity rate of CGM systems and target blood glucose levels (19). This discrepancy prompted the academic community to shift towards reconstructing intervention systems based on the core concept of patient empowerment. This transformation is reflected in several key aspects: remodeling patients’ cognitive behaviors through digital education modules—such as dynamic blood glucose mapping interactive learning—and virtual course educational interventions; implementing personalized education triggered by continuous monitoring data; and establishing a distributed nursing network supported by telemedicine policies (82, 83, 84). The essence of this shift lies in upgrading the paradigm from “technological tool substitution” to “activation of patient capacity.”

It suggests that the research paradigm is gradually shifting from an emphasis on technological applications toward enhancing patient engagement and prioritizing comprehensive lifestyle interventions. Looking ahead, academic inquiry into these emerging keywords is expected to deepen and expand, thereby facilitating the development of a more scientific, efficient, and accurate digital diabetes management system. This evolution will provide robust theoretical support and practical pathways for global diabetes prevention and treatment.

This study provides a comprehensive bibliometric analysis of research on digital diabetes management from 2010 to 2024. Going beyond conventional data analysis, we delineate the evolution of research hotspots, quantify geographical and institutional disparities, and synthesize recent advances and challenges related to AI and digital health, thereby offering an integrated overview of the knowledge structure and dynamic trends in this field. Our analysis highlights key internal and external factors shaping its future development: strengths include robust technological integration capabilities and growing real-world evidence; weaknesses involve persistent digital divides and algorithmic biases; opportunities are reflected in increasing policy support and progress in AI; while threats comprise data security risks and regulatory uncertainties. Importantly, we identify a paradigm shift from a purely technology-centric view toward a patient empowerment approach, and propose multi-stakeholder strategies to mitigate the digital divide. Furthermore, we critically examine the dual role of AI as both a facilitator and a potential amplifier of inequities, providing a balanced perspective on its opportunities and challenges in real-world applications. These findings offer valuable insights for researchers, practitioners, and policymakers aiming to advance equitable, effective, and sustainable digital diabetes management solutions worldwide.

## 5 Conclusion

With the profound integration of DHT and AI capabilities, the digital management of diabetes has markedly improved both the accuracy of disease monitoring and the timeliness of interventions. This advancement facilitates dynamic and personalized management plans tailored for diverse patient populations. Our study indicates that despite a continual increase in the prevalence of technological tools, significant population heterogeneity persists regarding glycemic target achievement and complication control. Future research should prioritize three key directions: first, developing intelligent decision-making systems that integrate multimodal data fusion to enable closed-loop interventions across biopsychosocial dimensions; second, constructing interpretable AI frameworks to address trust barriers between clinicians and patients caused by algorithmic black boxes; third, establishing multi-scale health economic evaluation models to provide evidence-based foundations for health insurance reimbursement policy formulation. Furthermore, our findings underscore several critical calls to action: at the academic level, priority should be given to investigating the coupling mechanisms between digital interventions and biological rhythms as well as assessing cross-cultural technology suitability. At the policy level, there is an urgent need to expedite the development of interoperability standards for DHTs alongside a governance framework for data sovereignty in order to facilitate global advancements in diabetes prevention and control systems.

## 6 Limitations

This study has several limitations. Firstly, the relevant articles were sourced exclusively from a single database, WOSCC, and the publication language was restricted to English. As bibliometric analysis relies on publications that were formally indexed by the database at the time of retrieval, some studies published after the search cut-off date or not yet fully indexed may have been excluded from the analysis. This may introduce selection bias and limit the comprehensiveness of the findings in representing the entire research landscape. Furthermore, the interpretation primarily concentrated on high-frequency and high-burst nodes, possibly neglecting other pertinent details. Additionally, researcher bias may be present, and despite considering all conceivable search terms, we cannot ensure the absence of omissions in the search results.

## Data Availability

The original contributions presented in the study are included in the article/Supplementary material, further inquiries can be directed to the corresponding author.
