# Supplementary material for: Digital management of diabetes global research trends: a bibliometric study
Source: Front Med (Lausanne). 2025 Oct 14;12:1620307. doi: 10.3389/fmed.2025.1620307 (PMC12558833; doi:10.3389/fmed.2025.1620307)
Supplement: Supplementary file 1 [file Data_Sheet_1.pdf]

Supplementary Material

Supplementary Figures

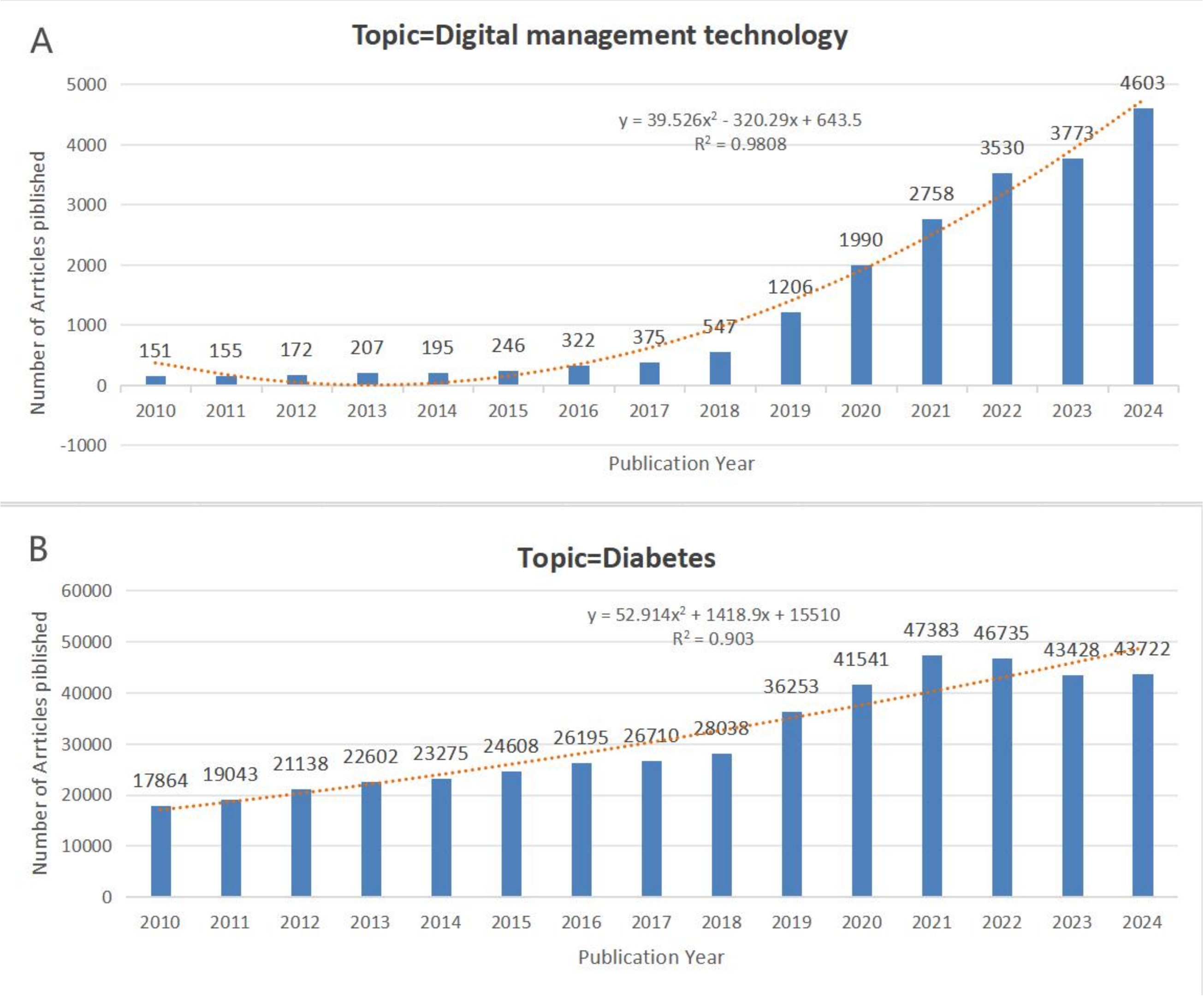

Supplementary Figure 1: (A) Trends in publication numbers within the field of digital management technologies from 2010 to 2024. (B) Trends in publication numbers within the field of diabetes from 2010 to 2024.
